# Enhanced Expression of Long Non-Coding RNA HOTAIR Is Associated with the Development of Gastric Cancer

**DOI:** 10.1371/journal.pone.0077070

**Published:** 2013-10-10

**Authors:** Hiroyuki Endo, Takeharu Shiroki, Takayuki Nakagawa, Misa Yokoyama, Keiichi Tamai, Hideaki Yamanami, Tsuneaki Fujiya, Ikuro Sato, Kazunori Yamaguchi, Nobuyuki Tanaka, Katsunori Iijima, Tooru Shimosegawa, Kazuo Sugamura, Kennichi Satoh

**Affiliations:** 1 Division of Cancer Stem Cell, Miyagi Cancer Center Research Institute, Natori, Miyagi, Japan; 2 Cancer Biology & Therapeutic, Miyagi Cancer Center Research Institute, Natori, Miyagi, Japan; 3 Department of Surgery, Miyagi Cancer Center, Natori, Miyagi, Japan; 4 Department of Pathology, Miyagi Cancer Center, Natori, Miyagi, Japan; 5 Molecular and cellular Oncology, Miyagi Cancer Center Research Institute, Natori, Miyagi, Japan; 6 Division of Gastroenterology, Tohoku University Graduate School of Medicine, Sendai, Miyagi, Japan; Peking University Cancer Hospital and Institute, China

## Abstract

The long non-coding RNA HOTAIR has been reported to be a poor prognostic biomarker in a variety of malignant tumors. However, little is known about the association of HOTAIR with gastric cancer. We examined the expression of HOTAIR in 68 gastric cancer samples using quantitative real-time RT-PCR and analyzed its correlation with the clinical parameters. The functional role of HOTAIR was examined by generating human gastric cancer cell lines with increased or suppressed HOTAIR expression. The anchorage -independent growth was assessed by soft agar assay. The increased or suppressed HOTAIR expressing gastric cancer cells were injected into the tail vein or peritoneal cavity of immunodeficient mice to examine the effect of this molecule on metastasis and peritoneal dissemination. The expression of HOTAIR was significantly higher in cancer lesions than in adjacent non-cancerous tissues in human gastric cancers. In the diffuse type of gastric cancer, the High-HOTAIR group (HOTAIR/GAPDH > 1) showed significantly more venous invasion, frequent lymph node metastases and a lower overall survival rate compared to the Low-HOTAIR group (HOTAIR/GAPDH < 1). Colony formation on the soft agar was enhanced in a HOTAIR-dependent manner. HOTAIR-expressing MKN74 formed more liver metastasis compared to control when they were injected into the tail vein of mice. In addition, reduced expression of HOTAIR in KATO III suppressed peritoneal dissemination. These results suggest that HOTAIR plays a pivotal role in the development of gastric cancer.

## Introduction

The incidence and mortality of gastric cancer have decreased dramatically over the past 50 years in most areas of the world, but it still remains the second leading cause of cancer-related deaths worldwide [1]. Despite recent advances in diagnostic techniques, such as magnifying endoscopy with narrow band imaging (NBI) [2], and in treatment including target therapy [3,4], there are still large numbers of gastric cancer patients with poor prognosis. The pathogenic mechanism contributing to the aggressive biological feature in this cancer remains to be clarified. 

The genome sequencing projects revealed that the human genome is comprised of less than 2% protein coding genes, and more than 90% of the genome is transcribed as non-coding RNAs (ncRNA) [5-7]. These ncRNAs are classified into two groups depending on the nucleotide size. Micro RNAs are approximately 18-25 nucleotides in length, while long non-coding RNA consists of more than 200 nucleotides. HOTAIR is a long non-coding RNA that was identified from a custom tilling array of the HOXC locus. HOTAIR was shown to trimethylate histon H3 lysin-27 of HOXD locus with the polycomb repressive complex 2 (PRC2) and inhibit HOXD gene expression, located on a different chromosome [8]. HOTAIR promotes metastasis through interaction with PRC2 to repress the transcription of multiple metastasis suppressor genes in breast cancer [9]. The enhanced expression of HOTAIR is associated with invasiveness, metastasis and poor prognosis in a variety of cancers such as breast, colon, pancreatic and lung cancer [9-12]. However, little is known about the expression and/or the function of HOTAIR in gastric carcinoma development. In the current study, we investigated the involvement of HOTAIR in human gastric cancer development and examined whether its expression would correlate with the aggressive behavior of gastric cancer.

## Materials and Methods

### Tissues

68 gastric cancer tissues were obtained from patients who underwent surgery at Miyagi Cancer Center (Natori, Japan), between 2007 and 2011. All samples were immediately frozen soon after resection in liquid nitrogen and stored at -80°C or fixed in 10% buffered formalin and embedded in paraffin wax. The gastric cancers were histopathologically classified as the intestinal type (n= 36), diffuse type (n=32) according to the classification of the World Health Organization and previous reports [13]. No patients received chemotherapy and radiotherapy before surgery. For statistical analysis, overall survival was defined by death from any cause, and Kaplan-Meier survival curves were used. 

### Cell lines

The gastric cancer cell lines MKN74 (intestinal type) [14] and KATO III (diffuse type) [15] were obtained from RIKEN BioResource Center (Tsukuba, Japan). Both cell lines were maintained in RPMI-1640 (Gibco/Life technologies Co.,CA) containing 10% inactivated FBS (EuroClone, Milano, Italy) with 100 units/mL penicillin and 100 μg/mL streptomycin (Gibco/Life technologies Co., CA) and cultured in a humidified 5% CO_2_ incubator at 37°C.

### RNA preparation, reverse transcription, and quantitative real-time PCR

Total RNA from frozen samples and cell lines was extracted by ISOGENE (NIPPON GENE, Tokyo, Japan) according to the manufacturer’s protocol. cDNAs from all samples were synthesized from 1.0 μg of total RNA by PrimeScript® 1st strand cDNA Synthesis Kit (TaKaRa Bio, Siga, Japan) following the manufacturer’s protocol. The expression of HOTAIR was quantified by LightCycler Brilliant SYBR Green qRT-PCR kit (Roche Applied Science,IN) following the manufacturer’s protocol with the specific primer sets according to the previous study [9]. The level of HOTAIR expression in each sample was normalized to the respective GAPDH expression level. The specificity of each PCR reaction was confirmed by melting curve analyses.

### HOTAIR expression retroviral vector

Human HOTAIR cDNA (addgene, Cambridge, MA) was amplified by PCR and was inserted into the pBabe puro vector (pBabe -HOTAIR). Recombinant retrovirus was produced with Platinum-A (Plat-A, Provided by Prof. Kitamura) packaging cell lines as described previously [16]. Briefly, Plat-A cells were transfected with pBabe -HOTAIR or pBabe-puro Vector (EV). Fugene-6 (Roche Applied Science) and Opti-MEM I (Gibco/Life technologies Co.) were added following the manufacturer's protocol. Forty-eight hours after transfection, the retrovirus-containing supernatant was collected and passed through a 0.45 μm filter. MKN74 cells were infected with the recombinant retroviruses and then selected with puromycin. 

### RNA interference

To knockdown HOTAIR in KATO III, we used Knockout™ RNAi Systems (Clontech Laboratories, Inc., Mountain View, CA) according to the manufacturer’s protocol. Briefly, we designed four shRNA sequence targeted HOTAIR as shown in Table 1. After annealing of the complementary shRNA oligonucleotides, we ligated the annealed oligonucleotides into pSIREN vector (shHOTAIR). Then, we transfected Plat-A cells with shHOTAIR or pSIREN Vector (EV), and infected KATO III with the recombinant retroviruses and selected with puromycin. The cells transduced with the sh-HOTAIR2 and -3 vector were chosen for further study. Each *in vitrto* experiment was performed in triplicate, repeated three times and representative data are shown. 

**Table 1:** Sequences of shRNA inserts for the shHOTAIR expressing vector.

shRNA	Sequence
shHOTAIR-1	
sense	gatccgaacgggagtacagagagattttcaagagaaatctctctgtactcccgttcttttttg
anti-sense	aattcaaaaaagaacgggagtacagagagatttctcttgaaaatctctctgtactcccgttcg
shHOTAIR-2	
sense	gatccgccacatgaacgcccagagattttcaagagaaatctctgggcgttcatgtggttttttg
anti-sense	aattcaaaaaaccacatgaacgcccagagatttctcttgaaaatctctgggcgttcatgtggcg
shHOTAIR-3	
sense	gatccgtaacaagaccagagagctgttttcaagagaaacagctctctggtcttgttattttttg
anti-sense	aattcaaaaaataacaagaccagagagctgtttctcttgaaaacagctctctggtcttgttacg
shHOTAIR-4	
sense	gatccaattcttaaattgggctggttcaagagaccagcccaatttaagaattttttttg
anti-sense	aattcaaaaaaaattcttaaattgggctggtctcttgaaccagcccaatttaagaattg

### Cell growth assay

1 x 10^4^ cells were seeded per well in 96 well plates in normal cell growth media. The 3-(4,5-dimethylthiazol-2-yl)-2,5-diphenyltetrazolium bromide, yellow tetrazole (MTT)　assay was performed using a Cell proliferation Kit I (GE Healthcare Life Sciences, NJ) according to the manufacturer’s protocol. Measurements of absorbance at 570 nm by VersaMax (Molecular Devices, CA) were made to estimate MTT-formazan production after 24, 48 and 72 hours incubation. The index was evaluated at 48 and 72 hours normalized to that at 24 hours.

### Soft agar colony formation assay

Soft agar assays were constructed in 6-well plates. The base layer of each well consisted of 1ml with final concentrations of 1 x media (RPMI-1640 plus 10% inactivated FBS) and 0.6% low melting point agarose. Plates were chilled at room temperature until solid, at which point a 1-ml growth agar layer was poured, consisting of 1 x 10^4^ cells suspended in 1 x media and 0.3% low melting point agarose. Plates were again chilled at room temperature until the growth layer congealed. A further 1 ml of 1 x media without agarose was added on top of the growth layer on day 0 and again on day 14 of growth. Cells were allowed to grow at 37°C for 4 weeks and total colonies were counted.

### Animals

6-week-old female NOD/Shi-scid-IL2Rγ ^null^ (NOG) mice were purchased from Central Institute for Experimental Animals (CIEA, Kawasaki, Japan) and used in this experiment. They were allowed to acclimate for a week before the experiments. They were housed in a clean room of the animal care facility at Miyagi Cancer Research Center and kept under standard temperature, humidity and timed lighting conditions and provided mouse chow/water ad libitum. 

### Tail vein assay of cancer cell metastasis

To evaluate the effect of HOTAIR expression on blood-borne metastasis, we injected 1.0 x 10^5^ cells of HOTAIR- (n=9) or EV-expressing (n=9) MKN74 with 0.2 ml PBS into the tail vein of mice. Mice were observed generally for signs of illness weekly for the length of the experiment, and they were sacrificed 8 weeks after injection. The lungs, livers, kidneys, adrenal glands, and spleens of the mice were excised and were investigated macroscopically about the presence of metastasis. 

### Peritoneal metastasis

1.0 x 10^6^ cells of shHOTAIR- (n=5) or EV-expressing (n=5) KATO III were injected with 0.2 ml PBS into the peritoneum of mice to evaluate the effect of HOTAIR expression on peritoneal metastasis. Mice were observed generally for signs of illness weekly for the length of the experiment, and they were sacrificed 8 weeks after injection. The presence of nodules and ascites in the peritoneal cavity was investigated.

### Histology and Immunohistochemistry

The excised tissues were fixed in 10% buffered formalin for 24 hours, then cut and embedded in paraffin for microscopic investigation. The samples were cut into 4-μm-thick sections and stained with hematoxylin and eosin (H&E). We investigated the presence of micro-metastasis that was not recognized macroscopically. The recognized tumors were further investigated immunohistochemically using primary antibody for human cytokeratin (Anti Cytokeratin Low, DC10/5D3, Nichirei Co, Tokyo, Japan) to make sure that they were derived from injected human gastric cancer cell. After deparaffinization, the tissue sections were incubated in methanol with 0.3% hydrogen peroxide in distilled water for 20 minutes to block the endogenous peroxidase activity. Antigen retrieval was performed by pressure cooking at 95°C for 5 min in 10 mM citrate buffer, pH 6.0, and then left to cool at room temperature for 30 minutes. Endogenous biotin- or avidin-binding sites were blocked by sequential incubation for 30 minutes. The sections were incubated overnight at 4°C with the primary antibody described above and were reacted with the second antibody. An avidin-biotin- peroxidase complex was used to detect the second antibody. The staining was visualized with diaminobenzidine (DAB). The sections were counterstained with hematoxylin, washed and dehydrated using a gradient of ethanol to xylene.

### Statistical analysis

The correlation of HOTAIR expression with the patient’s clinicopathological variables and the rate of metastasis in the injected mice were analyzed by the chi-square test. Statistical significance of differences between 2 groups was determined using Wilcoxon test and *P* value (

< 0.05) was regarded as statistically significant. Statistical difference between 3 groups was evaluated using Steel Dwass test or Bonferroni analysis and *P* value (< 0.017) was regarded as statistically significant Overall survival probability was analyzed by the Kaplan-Meier methods and evaluated by log-rank test, and *P* value (< 0.05) was regarded as statistically significant. All statistical analyses were performed using Ekuseru-Toukei 2012 (Social Survey Research Information Co., Ltd., Tokyo, Japan). The *P* value (< 0.05) was regarded as statistically significant.

### Ethics

 The Institutional Review Board of the Miyagi Cancer Center (MCC) approved this study protocol, and written informed consent was obtained from each patient (Permit Number: MCC-22-30). The protocol of animal experiments was approved by the MCC Animal Care and Use Committee (Permit Number: MCC-AE-2011-12).

## Results

### The HOTAIR expression of human gastric cancer samples

**Figure 1:**
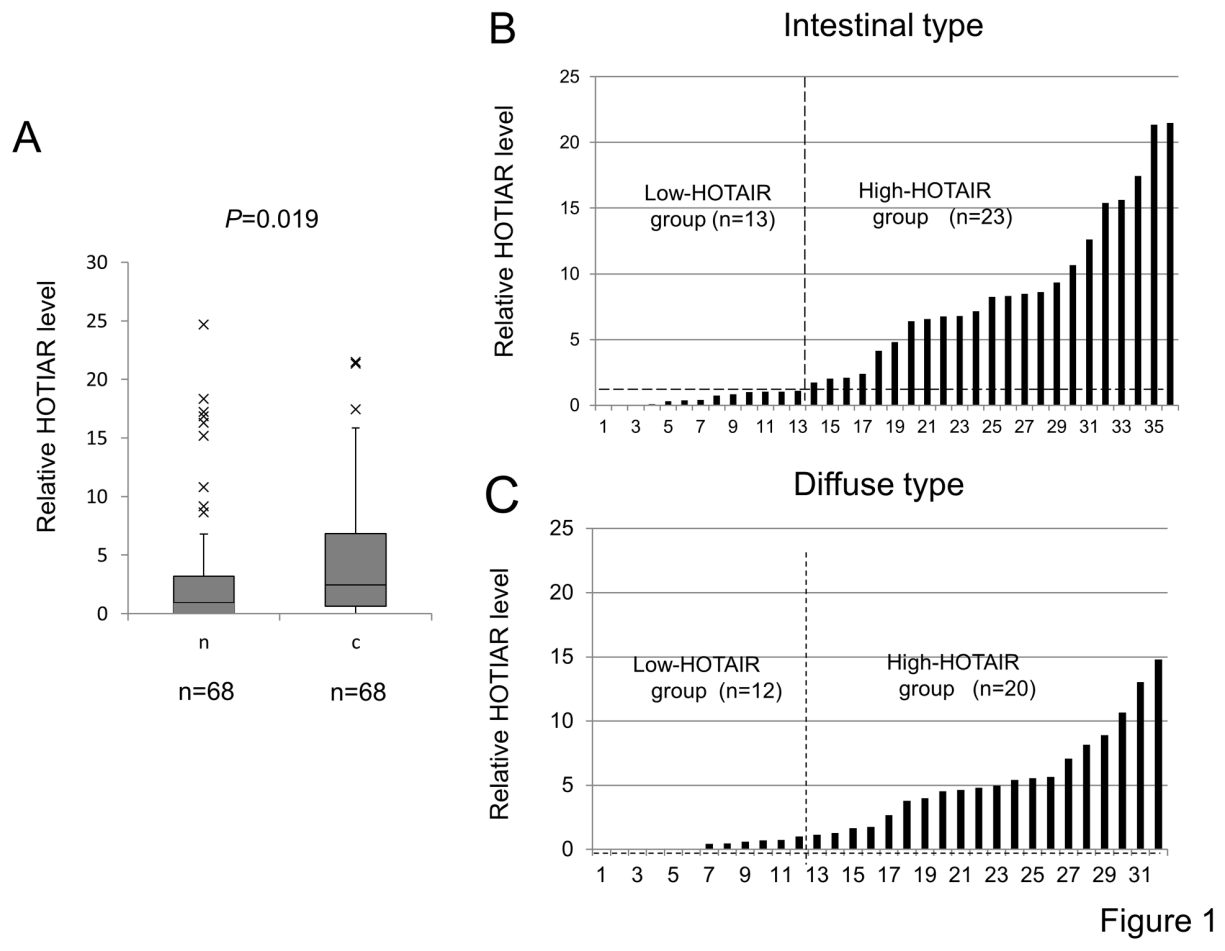
The expression of HOTAIR in human gastric carcinoma tissues. **A**, HOTAIR expression levels of cancerous and adjacent noncancerous tissues from the gastric cancer patients were examined by qRT-PCR and normalized by the GAPDH expression level. The expression levels of HOTAIR were significantly higher in carcinoma lesions compared to those of non-cancerous lesions (*P*= 0.019). **B** and **C**, The human gastric cancer tissues were further classified according to the HOTAIR expression level into a High-HOTAIR group (HOTAIR / GAPDH >1.0) and Low-HOTAIR group (HOTAIR /GAPDH < 1.0) in the intestinal (B) and diffuse type (C) of gastric cancer, respectively.

The HOTAIR expression levels of cancerous and adjacent noncancerous tissues from the gastric cancer patients were examined by qRT-PCR and normalized by the GAPDH expression level. The mean expression level of HOTAIR was 4.952±6.462 and 3.804 ± 6.721 (HOTAIR/GAPDH ± standard deviation) in carcinoma and non-cancerous lesions, respectively. The expression levels of HOTAIR were significantly higher in carcinoma lesions compared to those of non-cancerous lesions (*P*=0.019, Figure 1A).

### Association between the expression of HOTAIR and the clinicopathological factors in human gastric cancers

**Figure 2:**
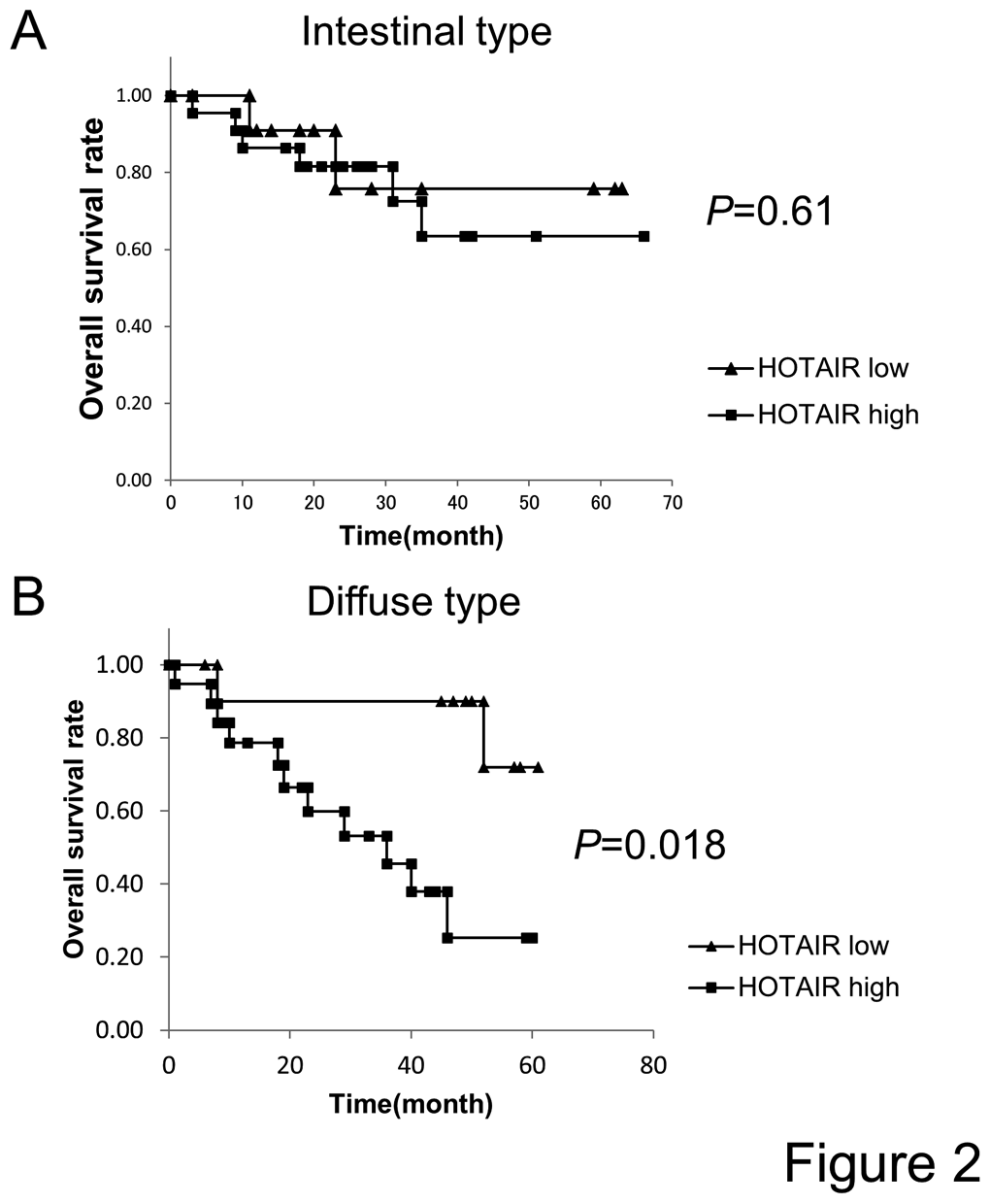
The correlation between HOTAIR expression level and prognosis of gastric cancer patients. **A**, In the intestinal type of gastric cancer, no significant relation was seen between the HOTAIR expression and overall survival, although the High-HOTAIR group tended to show shorter survival than the Low-HOTAIR group. **B**, the High-HOTAIR group showed significantly shorter survival than the Low-HOTAIR group in the diffuse type of gastric cancer (*P*=0.018).

The human gastric cancer tissues were histopathologically classified into intestinal (n=36) and diffuse types (n=32). The human gastric cancer tissues were further classified according to the HOTAIR expression level into the High-HOTAIR group (HOTAIR / GAPDH >1.0) and Low-HOTAIR group (HOTAIR /GAPDH < 1.0) (Figures 1B and C). The association of HOTAIR expression with the clinicopathological features in the intestinal and diffuse type of gastric cancer is summarized in Tables 2 and 3, respectively. In the intestinal type of gastric cancer, no significant correlation was found between HOTAIR expression and the clinicopathological findings (Table 2). In addition, no significant relation was seen between HOTAIR expression and the overall survival, although the High-HOTAIR group tended to show shorter survival than the Low-HOTAIR group (Figure 2A). On the other hand, a significant association was found between HOTAIR expression and lymph node metastasis (P= 0.043), venous infiltration (*P*=0.0083), and short overall survival (*P*=0.018) in the diffuse type gastric cancer (Table 3 and Figure 2B). However, there was no association between the stage and HOTAIR expression. 

**Table 2:** The association of HOTAIR expression with clinicopathological features in intestinal type of gastric cancer.

	HOTAIR low	HOTAIR high	*P* value
Age	73.07±9.18	70.87±10.65	0.53
Gender			0.63
Male	10	16	
female	3	7	
T			0.87
T1, T2	6	10	
T3, T4	7	13	
N			0.26
N0	7	8	
N1,N2,N3	6	15	
Ly			0.68
ly0	6	9	
ly1,ly2,ly3	7	14	
v			0.19
v0	8	9	
v1,v2,v3	5	14	
Stage			0.50
1,2	7	15	
3,4	6	8	0.50

**Table 3:** The association of HOTAIR expression with clinicopathological features in diffuse type of gastric cancer.

	HOTAIR low	HOTAIR high	*P* value
Age	59.250±10.75	66.35±10.16	0.07
Gender			0.71
male	8	12	
female	4	8	
T			0.87
T1, T2	1	2	
T3, T4	11	18	
N			0.043*
N0	8	6	
N1,N2,N3	4	14	
ly			0.40
ly0	6	7	
ly1,ly2,ly3	6	13	
v			0.0083*
v0	11	9	
v1,v2,v3	1	11	
Stage			0.31
1,2	7	8	
3,4	5	12	

* Statistically significant.

### Generation of HOTAIR up- and down-regulated cells and correlation with cell viability

**Figure 3:**
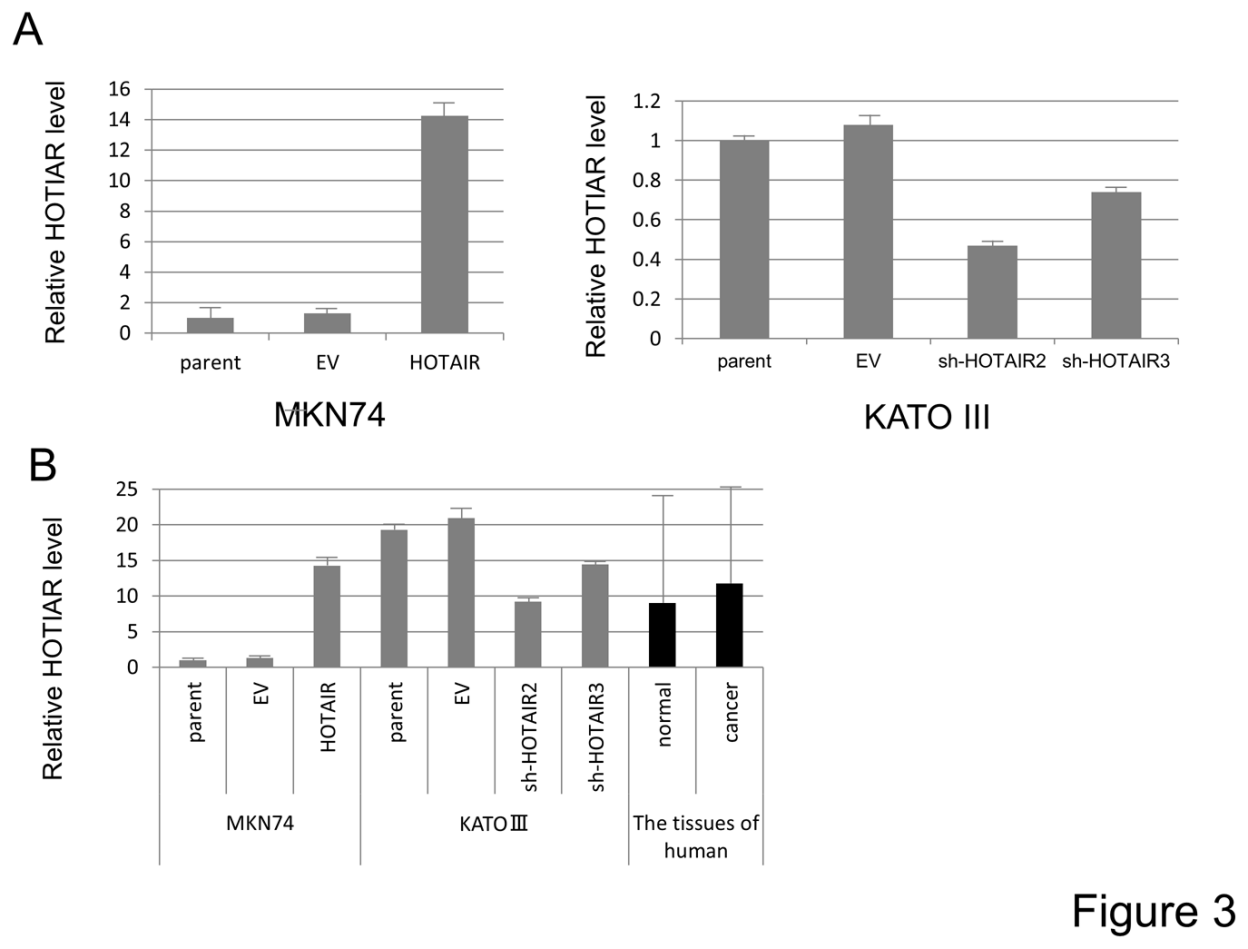
Generation of increased and decreased HOTAIR expressing gastric carcinoma cells and compared relative expression between cell lines and human gastric tissues. **A**, Relative HOTAIR expression levels in gastric cancer cell lines were analyzed by qRT-PCR analysis. MKN-HOTAIR cells expressed about 10 fold higher levels of HOTAIR than parental MKN74 cells and MKN-EV. On the other hand, the expression of HOTAIR in KATO-shHOTAIR cells was reduced by 30 to 70% compared to parental KATO III and KATO-EV cells. **B**, The bar graph demonstrated the relative expression of HOTAIR in gastric cancer cell lines, normal and carcinoma tissues of the stomach analyzed in this study. Expression of HOTAIR was normalized to that of GAPDH. Values are expressed relative to 1.00 for expression in parental MKN74 cells.

To assess the functional role of HOTAIR in gastric cancer development, we generated stably HOTAIR expressing MKN74 cells (MKN-HOTAIR) and stably shHOTAIR expressing KATO III cells (KATO-shHOTAIR). qRT-PCR analysis revealed that MKN-HOTAIR cells expressed a level of HOTAIR about 10 fold that of EV expressing (MKN-EV) or parental MKN74 cells and that the expression of this molecule was reduced by 30 to 70% in KATO-shHOTAIR cells compared to EV transduced (KATO-EV) or parental KATO III (KATO-EV) cells (Figure 3A). The results of the MTT assay demonstrated that neither up-regulation nor down-regulation of HOTAIR affected gastric cancer cell proliferation (Figure S1).

### Soft agar colony formation assay

**Figure 4:**
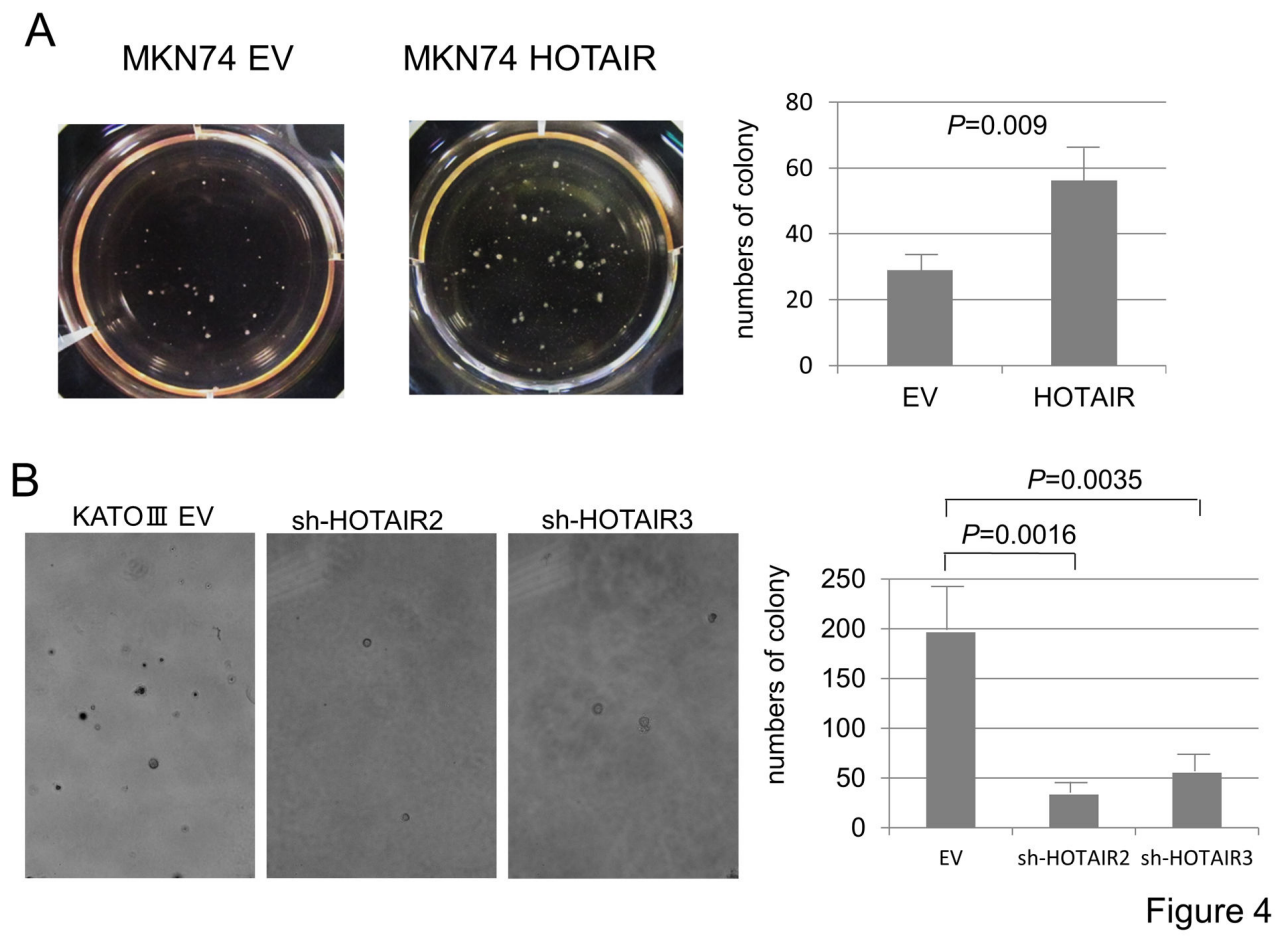
Anchorage-independent cell growth in HOTAIR expressing or down-regulated gastric carcinoma cells. Potential of anchorage-independent growth of gastric cancer cells was assessed by soft agar assay. MKN-HOTAIR cells formed significantly larger numbers of colonies than MKN-EV cells (*P*=0.009) (**A**), while KATO-shHOTAIR cells showed significantly fewer colonies than KATO-EV cells in HOTAIR expression manner (EV versus sh- HOTAIR2, *P*= 0.016 and EV versus sh-HOTAIR3, *P*= 0.0035) (**B**).

To elucidate the functions of HOTAIR in the anchorage-independent growth of gastric cancer cells, we utilized a soft agar assay. MKN-HOTAIR cells formed a larger number of colonies than MKN-EV cells (*P*=0.009, Figure 4A). Consistent with this finding, KATO-shHOTAIR cells showed significant fewer colonies than KATO-EV cells (Figure 4B). In addition, the gastric cancer cells with similar HOTAIR expression level (MKN-HOTAIR and shHOTAIR-3, Figure 3B) formed similar numbers of colonies, indicating that anchorage-independent growth of gastric cancer cells was regulated in a HOTAIR-dependent manner.

### HOTAIR expression and gastric cancer cell metastasis and peritoneal dissemination

**Figure 5:**
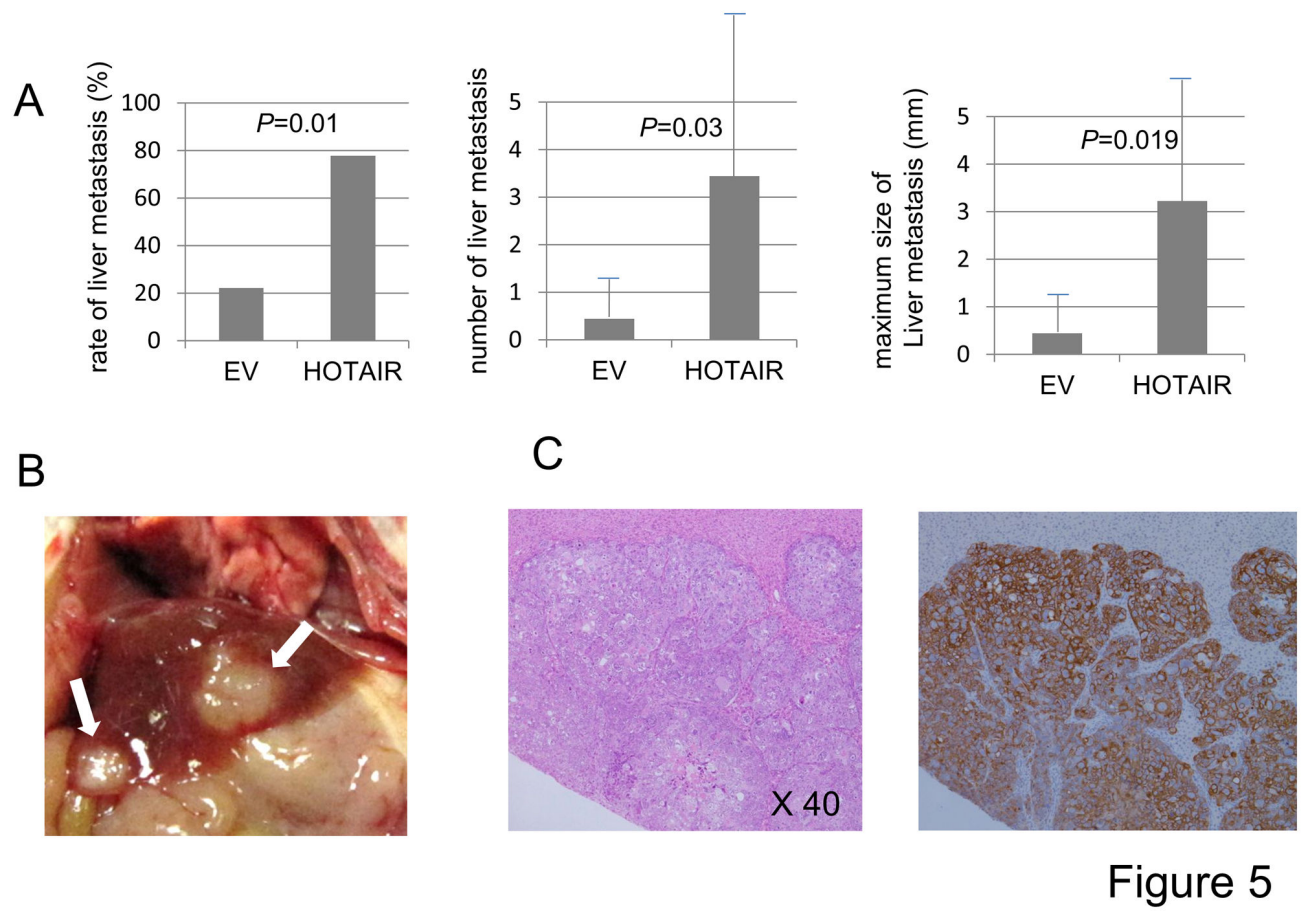
The association of HOTAIR expression with liver metastasis. **A**, The effect of HOTAIR on metastasis was investigated by tail vein assay. 1.0 x 10^5^ of HOTAIR expressing cells (MKN-HOTAIR, n=9) and control cells (MKN-EV, n=9) were injected into the tail vein of mice. MKN-HOTAIR cells more frequently showed liver metastases (*P*=0.01) compared to MKN-EV cells (left panel). In addition, the numbers (middle panel) and sizes of the metastatic liver tumors (right panel) were significant larger with MKN-HOTAIR cells than with MKN-EV cells (*P*=0.03 and *P*=0.019, respectively). **B**, The metastatic tumors could be clearly recognized macroscopically (arrow). **C**, The tumor cells formed glands and were thought to be derived from the injected human gastric cancer cell lines (left panel, HE, orginal magnification x40). These tumor cells showed intense immunoreactivity for human cytokeratin (right panel).

**Figure 6:**
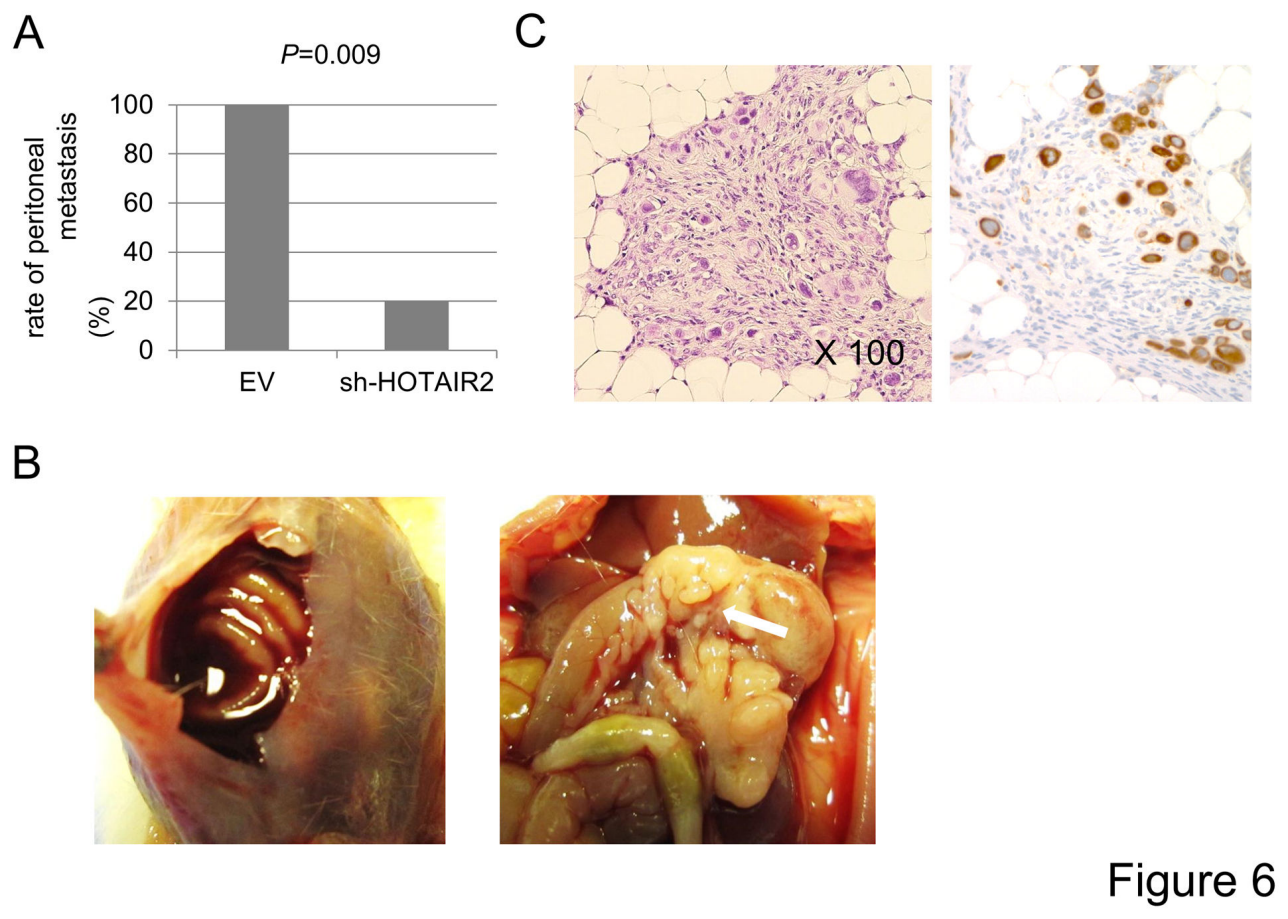
HOTAIR expression and the peritoneal dissemination of gastric carcinoma cells. **A**, Peritoneal dissemination was significantly reduced (1/5, *P*=0.009) when HOTAIR was inactivated in KATO III cells by shRNA transduction, while all control cells formed peritoneal dissemination (5/5). **B**, The mouse with peritoneal dissemination showed bloody ascites (left panel) and nodules (arrow in right panel) in the peritoneal cavity. **C**, The nodules in the peritoneal cavity consisted of cancer cells, and some of them had mucin like a signet ring cell carcinoma. They were thought to be derived from the injected human gastric cancer cell lines (HE, original magnification x 100) since these cells showed intense immunoreactivity for human cytokeratin (right panel) .

Since HOTAIR enhanced the anchorage-independent but not anchorage-dependent growth of gastric cancer cells, we hypothesized that HOTAIR might contribute to distant metastasis and/or peritoneal dissemination rather than direct invasion to neighboring organs. To address this issue we employed a tail vein assay and peritoneal injection of cells to examine whether HOTAIR expression promotes blood -borne metastasis and peritoneal dissemination, respectively. As expected, MKN-HOTAIR cells frequently exhibited liver metastases (*P*=0.01) compared to MKN-EV cells. In addition, the numbers and sizes of the metastatic liver tumors were significant larger with MKN-HOTAIR cells than with MKN-EV cells (*P*=0.03 and *P*=0.019, respectively, Figures 5A and B). Interestingly, one mouse injected with MKN-HOTAIR showed metastasis to the kidney and the adrenal gland in addition to the liver, while with MKN-EV cells metastatic tumors formed only in the liver. Consistently, peritoneal dissemination was significantly reduced (1/5, *P*=0.009) when HOTAIR was inactivated in KATO III cells while all control cells formed peritoneal dissemination (5/5) (Figures 6A and B). The metastatic tumors were histologically confirmed and they showed intense positive staining for human cytokeratin (Figures 5C and 6C).

## Discussion

In this study, we investigated the expression of HOTAIR in gastric cancer and examine its correlation with the clinicopathological features including patients’ prognosis. Our results clearly revealed enhanced expression of HOTAIR in cancer tissues compared to non-cancerous tissues of the stomach and that the high level expression of this molecule was associated with lymph node metastasis, venous invasion and poor survival in the diffuse type of gastric cancer. These findings are consistent with the recent study showing that up-regulation of HOTAIR is found in gastric cancer compared to normal tissues and is correlated with the tumor stage and lymph node metastasis [17]. In addition, HOTAIR-expressing gastric cancer cells exhibited the enhancement of anchorage-independent cell growth *in vitro* and metastases to the liver *in vivo* while reduced expression of HOTAIR in gastric cancer cells resulted in decreased anchorage-independent growth and peritoneal dissemination, suggesting that HOTAIR plays a pivotal role in gastric cancer progression. 

Higher expression of HOTAIR was detected in cancer cells than in the corresponding non-tumor cells in various cancers including breast, colon, liver, pancreas and nasopharyngeal cancer [9-11,18-20]. In these cancers, HOTAIR was also shown to be associated with metastasis and/or poor prognosis. Furthermore, high level of HOTAIR expression was correlated with short disease-free survival in lung cancer [12]. Therefore, our present findings are consistent with previous studies indicating that the expression of HOTAIR is involved in the enhanced aggressiveness of various types of carcinoma cells. On the other hand, we could not find negative prognostic effect of HOTAIR in the intestinal type of gastric cancer, although the High-HOTAIR group tended to show shorter overall survival compared to the Low-HOTAIR group. This might be due to the small number of patients in the current study, since forced expression of HOTAIR in an intestinal gastric cancer cell line (MKN74) gained the aggressive phenotype *in vitro* and *in vivo*. Further investigations with large numbers of samples in addition to the experimental study using other intestinal gastric cancer cell lines would be required to address this issue.

In the current study, no association was found between HOTAIR expression and cell growth in gastric cancer. In breast, colon and liver cancer, the involvement of HOTAIR in cell growth has not been demonstrated [9,10,18], whereas HOTAIR promoted and reduced cell proliferation in pancreatic cancer [11] and lung cancer [12], respectively. Further, no significant difference was found in subcutaneous tumor growth in NOG mice between HOTAIR-expressing and control cells in the current study (data were not shown). Based on these findings, it is likely that the link between HOTAIR expression and cell proliferation is dependent on the type of cancer. In contrast, the anchorage-independent growth of gastric cancer cells was clearly enhanced in a HOTAIR-dependent manner. In addition, HOTAIR expression caused more metastases to the liver and other organs, and a defect of HOTAIR suppressed the peritoneal dissemination of gastric carcinoma cells. These observations, together with the fact that HOTAIR expression contributes to metastasis in various carcinomas [9-12,18], suggest that HOTAIR is mainly involved in the metastatic process rather than in the growth of primary tumor. 

In conclusion, enhanced HOTAIR expression in the diffuse type of gastric cancer was associated with the incidence of venous invasion and poor prognosis independently of T factor, and forced expression of HOTAIR in gastric cancer cells promoted anchorage-independent cell growth *in vitro* and metastases to liver *in vivo*. These results indicate that HOTAIR is likely to be involved in the development of gastric cancer and might also be a therapeutic target in the treatment of gastric cancer. 

## Supporting Information

Figure S1 **The association of cell proliferation with HOTAIR expression.** The results of MTT assay evaluated at 72 hours were normalized to that at 24 hours. No relation was found between HOTAIR expression and gastric cancer cell proliferation.
(TIF)
