# Supplementary figures and images for: Enhanced Expression of Long Non-Coding RNA HOTAIR Is Associated with the Development of Gastric Cancer
Source: PLoS One. 2013 Oct 10;8(10):e77070. doi: 10.1371/journal.pone.0077070 (PMC3795022; doi:10.1371/journal.pone.0077070)

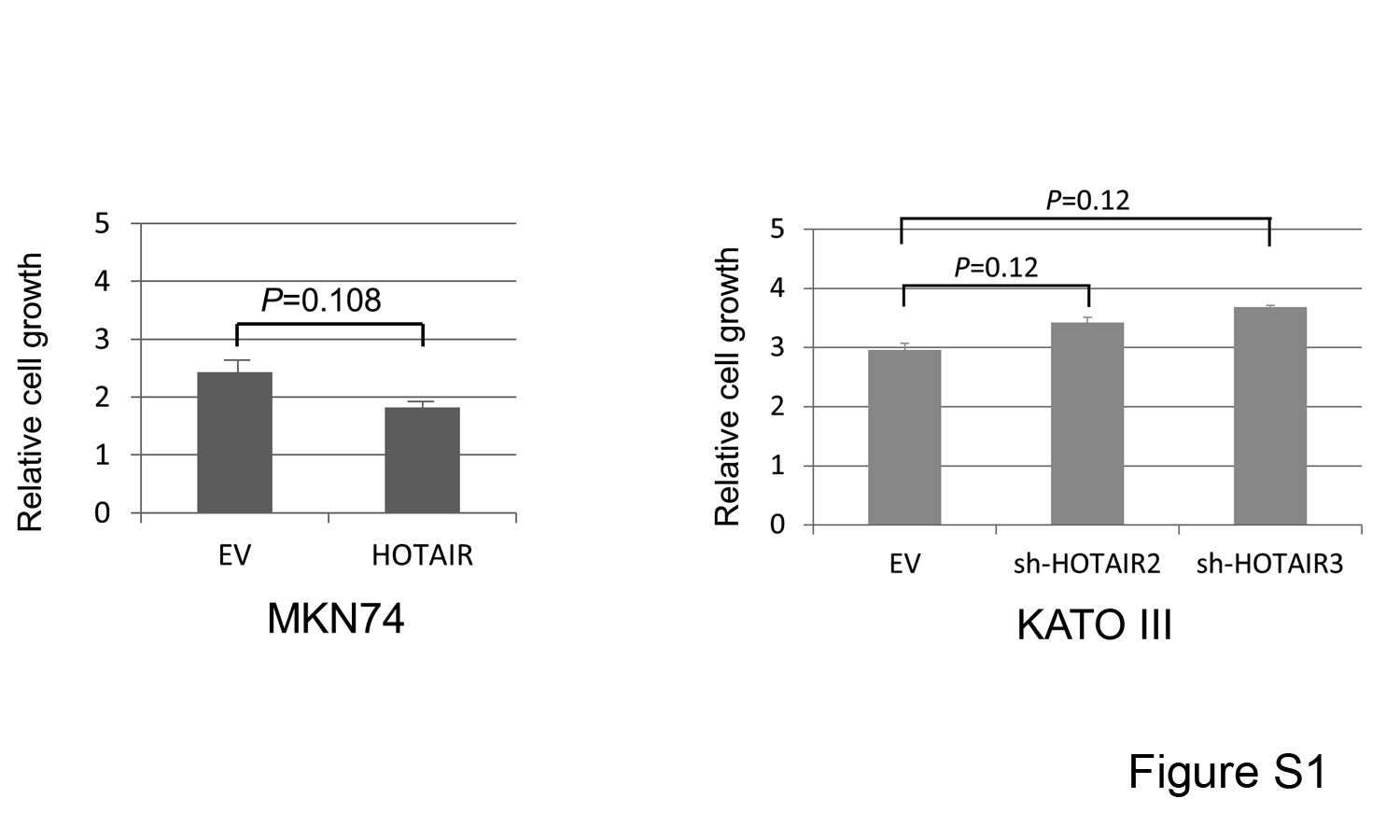

Supplement: Figure S1 — The association of cell proliferation with HOTAIR expression. The results of MTT assay evaluated at 72 hours were normalized to that at 24 hours. No relation was found between HOTAIR expression and gastric cancer cell proliferation. (TIF) [file pone.0077070.s001.tif]
